# Titanium surface interacting with blood clot enhanced migration and osteogenic differentiation of bone marrow mesenchymal stem cells

**DOI:** 10.3389/fbioe.2023.1136406

**Published:** 2023-05-16

**Authors:** Jia Li, Juan Zhao, Yangbo Xu, Antian Xu, Fuming He

**Affiliations:** Department of Prosthodontics, Stomatology Hospital, School of Stomatology, Zhejiang University School of Medicine, Zhejiang Provincial Clinical Research Center for Oral Diseases, Key Laboratory of Oral Biomedical Research of Zhejiang Province, Cancer Center of Zhejiang University, Hangzhou, China

**Keywords:** dental implants, blood clot, blood incubation, bone marrow mesenchymal stromal cells, osteogenesis

## Abstract

**Introduction:** Blood clot formation is the initial phase upon implantation, and the feature of blood clot orchestrates the following complement system activation, coagulation cascade, and bone marrow mesenchymal stromal cells (BMSCs) recruitment. This study aimed to investigate the effect of implant surface on blood-material interactions and subsequent BMSC cellular behaviors.

**Methods:** This study was established to imitate the physiological process of implantation *in vivo* and *in vitro*. Whole blood was incubated with polished titanium (PT) surfaces and sandblasted and double acid-etching (SLA) surfaces for 10 min or 2 h, then seeded with BMSCs. The adhesion, proliferation, migration, and differentiation of cells were studied at specific time points. Titanium implants were implanted into the tibia in vivo and were screwed out after implantation. The activation of the coagulation cascade, platelets, complement system, and clot networks were assessed and further quantitatively analyzed.

**Results:** Compared with the PT surface, the SLA surface induced the earlier and stronger blood coagulation cascade and formed a more stratified clots network with fibrinogen, platelets, and CD14 positive cell. The adhesion, proliferation, and migration of BMSCs were enhanced by pre-incubated surfaces. The higher levels of the osteogenic-related genes, ALP activity, and calcium nodule formation were showed on SLA surfaces with blood incubation.

**Conclusion:** SLA titanium surfaces play a role in influencing the formation of blood clots and coordinating surface-blood interactions and cell biological processes. These findings provide the idea of modifying the blood clots formed on the implant surface by biomaterials modification and thus has implications for the development of better osteogenic biomaterials.

## 1 Introduction

Biomaterials are commonly used to repair damaged or missing tissue, and implants are the gold standard for dental replacement. Implant integration is a multi-temporal process mediated by numerous molecular, cellular, and immune cascades (Trindade et al., 2016). It is well known that the surface properties of materials can influence the clinical outcome of implants, and materials can even regulate cell behavior by directly altering the initial blood clot structure and protein adsorption (Yang and Xiao, 2020).

As blood clot formation is the initial and foremost phase upon implantation, and the feature of blood clot orchestrates the following provisional matrix around the biomaterial surface, causing further activation and aggregation of platelets and the division of fibrinogen into fibrin via thrombin (Gorbet and Sefton, 2004; Wei et al., 2018). Meanwhile, complement proteins, activated upon contact with biomaterials, immediately facilitate platelets adhesion and activation that further propagate the coagulation cascade, the complement system activation, and immune cell recruitment. The provisional matrix releases a myriad of pluripotent factors, chemokines, and growth factors, and provides an interlaced scaffold for inflammatory, mesenchymal cells, and osteogenic cells (Wang et al., 2016; Huang et al., 2019).

The thickness and density of the clot network are affected by the miscellaneous surface physicochemical modifications (Fan et al., 2017; Bai et al., 2021). To date, nanostructured titanium surfaces perform better osseointegration due to continuous protein adsorption and dense blood clot (Lukaszewska-Kuska et al., 2018). The clot features are modulated by arrays of titanium dioxide nanotubes with distinct nano-diameters, which induce macrophages to polarize to the M2 phenotype and lead to a favorable immune response (Kopf et al., 2015a). Also, blood-derived products and blood pre-fabricated scaffolds have been trialed as potential bioactive materials to enhance bone regeneration (Melville et al., 2019). Accordingly, a clot network with appropriate structure is gradually considered an alternative nature healing scaffold to accelerate bone repair and integration. The previous studies usually conducted experiments *in vitro*, and the complexity of the *in vivo* environment makes the formation and features of clots on the material surface with distinct characteristics, which have not been well-understood. Besides, most of these studies have focused on the role of single blood components, other than the complex composition of whole blood.

In addition, moderate roughness and hydrophilic surfaces have better osteogenic properties, dues to the enhancement of the adhesion, proliferation, and osteogenic differentiation of cells (Faia-Torres et al., 2014; Huang et al., 2019; Xing et al., 2020). However, current studies mainly focused on the direct interactions between biomaterials and cells and neglected the physiological involvement of the blood and interstitial fluids. The role of the clot network formed immediately between surface properties and bone marrow stromal cells (BMSCs) biological behaviors is still unclear.

Our study utilized the fresh whole blood pre-incubated titanium surfaces *in vitro* to investigate the synergic effect of the clot network on the adhesion, migration, and osteogenic differentiation of BMSCs. Further, the physiological processes of implantation placement were simulated *in vivo* to assess platelet activation, coagulation cascade, complement systems, and cellular responses, and to analyze the adsorbed proteins and clot features. This study is to explore the effect of surface properties on early bone healing and osteogenesis: 1) whether titanium surface roughness and hydrophilicity affect the platelet activation and the clot features; 2) if the clot features steer the recruitment and osteogenic differentiation of BMSCs.

## 2 Materials and methods

### 2.1 Sample preparation and characterization

The commercially pure titanium slices (10 mm in length, 1 mm in thickness for *in vitro* experiments) and screw-shaped titanium implants (Ø 2 mm × 4 mm for *in vivo* studies) were prepared by Zhejiang Guangci Medical Appliance Co., Ltd., Ningbo, China. Then, the samples were divided into two groups: 1) the experimental group with sandblasted and double-acid etched titanium (SLA) surface was prepared as previously described (He et al., 2009). 2) the control group with polished titanium (PT) surface was sequentially polished with 280-, 600-, and 1200-grid silicon carbide papers (CarbiMet, Buehler). Before the following procedures, all samples were sterilized by UV irradiation.

The surface microstructure of the samples was observed using a field-emission scanning electron microscope (FE-SEM, SU8010, Hitachi, Japan). The static contact angle was measured as the assessment of hydrophobicity via Contact Angle Meter (JC 2000C, POWEREACH, China). A liquid drop of deionized water was deposited on the flat surface under the same environmental condition. The contact angle with water was measured from photographs using ImageJ software. The roughness of samples was evaluated by the arithmetic average of the absolute values of the irregularity (Ra), the root means square of the roughness of the profile (Rq), and the maximum height of the profile (Rz) via surface rough-meter (NAHOSCPEIVA, Veeco, United States). Each measurement was repeated three times.

### 2.2 Isolation and culture of BMSCs

BMSCs were obtained from the femur of 4-week-old male Sprague-Dawley (SD) rats. The bone marrow of the femoral midshaft was flushed out and suspended in alpha-modified eagle medium (α-MEM, Hyclone) supplemented with 10% fetal bovine serum (FBS, Hyclone) and 100 U/mL of penicillin and streptomycin. The cells were incubated at 37°C in a 95% humidified atmosphere with 5% CO_2_. Passages three to five of cells were used in the following *in vitro* experiments.

### 2.3 Blood incubation of titanium surface

To mimic the initial contact between implant surfaces and fresh blood during surgery, pre-incubated titanium slices were prepared. The whole blood of healthy male SD rats was collected with the blood collection tube with 10 IU heparin (Solarbio). Each slice was transferred into a 24-well plate using sterile forceps and incubated in 500 μL fresh rat blood for 10 min or 2 h at 37°C. The plates were placed on the table concentrator with steady shaking at 10 rpm to avoid blood sedimentation. After gently washing and removing non-adsorbed components, some titanium slices were collected as samples for the following assays. The other titanium slices were immersed in normal mediums for 6 h, then the supernatants were collected as conditioned mediums for subsequent experiments.

### 2.4 Cell adhesion and proliferation assays

To evaluate the effect of the formation of clot network and the release of blood components on initial cell adhesion and proliferation, the BMSCs were seeded at an initial density of 2 × 10^4^ cells/mL on the control or pre-incubated titanium slices which were placed in the bottom of 24-well plates and cultured with Dulbecco’s modified eagle medium (DMEM, Hyclone) + 10% FBS. After 4 and 24 h culture, adherent cells were fixed with 4% paraformaldehyde (PFA, Solarbio) and stained with rhodamine-phalloidin (Cytoskeleton, Inc.) and 4′,6-diamidino-2-phenylindole dihydrochloride (DAPI). The cell morphology was observed and imaged by a laser confocal scanning microscope (LCSM, Nikon A1, Japan).

The proliferation of cells was detected by AlamarBlue assay (Invitrogen, Grand Island, NY) at 1, 3, and 7 days. Briefly, the cells were incubated on control or pre-incubated surfaces for 3 h in a medium complemented with 10% (v/v) AlamarBlue reagent. The optical density was valued (λex = 540 nm, λem = 590 nm) by a SpectraMax M5 (Molecular Devices, China). The results were analyzed by plotting optical density against cell concentration.

### 2.5 Scratch wound assays

A scratch wound assay was performed to study the influence of the pre-incubated titanium on BMSCs migration. BMSCs were seeded on SLA and PT slices in 24-well plates for 24 h to obtain monolayers and then a vertical scratch was made to generate an acellular region on each sample. After washing and removing the detached cells, each well was added 1 mL DMEM containing 5% FBS for 12 or 24 h. The F-actin and nuclei were labeled with rhodamine-phalloidin and DAPI. The movement of the cell to the acellular area of each sample was photographed under LCSM.

To further investigate the effects of the release of blood components on BMSCs mobility, the BMSCs were seeded in 6-well plates directly and grown to 75%–85% confluence in a normal medium. After vertically scratched to create a “wound” score, each well was covered with 2 mL conditioned medium as previously described for 12 and 24 h. Then the wounds were recorded by an inverted microscope (CKX41, Olympus, Japan). The wound healing rate was calculated by the percentage of the wound closure area at different time points using the ImageJ software.

### 2.6 Transwell assays

Transwell assay was conducted in the 24-well transwell chambers of 8 μm nitrocellulose pore filters (Corning-Costar, Kennebunk, United States). BMSCs with a density of about 3 × 10^5^ cells/mL were inoculated into the upper chamber (200 μL DMEM + 1% FBS), and 1 mL of conditioned mediums was added into the lower chamber. After incubation for 6 h, the cells penetrating the upper chamber membranes were fixed with 4% PFA and stained with 1% crystal violet dye solution (Saichuang Technology, Wuhan, China), and photographed by a microscope (CKX41, Olympus, Japan). The migration rate was quantified by counting the staining regions of five randomly selected fields on each transwell membrane.

### 2.7 Real-time quantitative PCR (RT-qPCR)

The RT-qPCR assay was performed to study the effect of different pre-incubated surfaces on the osteogenic and angiogenic potential of BMSCs at the gene level. After being cultured for 3 or 7 days, the total RNA of BMSCs was isolated and purified by an RNAeasy Mini kit (No.74106, Qiagen). The relative mRNA expression level of osteogenic genes [runt-related transcription factor 2 (*Runx2*), alkaline phosphatase (*Alp*), osteoprotegerin (*Opg*), and *osterix*] and angiogenic genes [vascular endothelial growth factor (*Vegf*) and platelet derived growth factor (*Pdgf*)] were determined by using an SYBR Green I kit (Takara, Osaka, Japan) on AppliedBiosystems ViiA 7 (Thermo Fisher Scientific, United States). Each sample was analyzed in triplicate, and the expression levels of target genes are shown relative to the housekeeping gene GAPDH as a control using the comparative Ct (2^−ΔΔCT^) method. All primer sequences are listed in Supplementary Table S1.

### 2.8 Osteogenic differentiation assays

To further assess the osteogenic differentiation of BMSCs, cells were seeded on Ti slices with or without pre-incubation. The osteogenic medium (OM: growth medium supplemented with 10 nM dexamethasone, 100 μM ascorbic acid, and 10 mM β-glycerophosphate) was changed twice per week. These cultures were used for alkaline phosphatase activity and alizarin red staining.

#### 2.8.1 Alkaline phosphatase activity

After 4 and 7 days, cells were lysed with 100 μL RIPA lysis buffer. The supernatant was collected after centrifugation of 12,000 rpm/min at 4°C for 15 min. The level of ALP activity was measured using an ALP Assay Kit (Wako, Osaka, Japan) according to the manufacturer’s instruction.

#### 2.8.2 Alizarin red staining and quantification

After 21 days, cell layers were washed with phosphate-buffered saline (PBS) and then fixed with 4% PFA for 15 min. Cells were stained with 0.2% alizarin red solution (Solaibio, Technology) for 20 min. Staining cells and calcium nodules were washed with double-dilution water and were photographed under a light microscope. For quantitative analysis of alizarin red of cell layers, 1 mL of 10% cetylpyridinium chloride (C129534, Aladdin, China) was added into each well. After the dissolution of calcium nodules, the optical density of solution was detected by a microplate reader at the wavelength of 590 nm.

### 2.9 Animals and surgical procedures

Animal experiments were approved by the Institutional Animal Care and Use Committee of Zhejiang University, Hangzhou, China (ZJU20190084). A total of 36 male SD rats weighing around 300 g were used. These rats were kept at a constant temperature and sterile operating table for implantation. In brief, SD rats were fixed on the bench and maintained under anesthesia by intraperitoneal injection of 10% chloral hydrate (0.33 mL/100 g, C104202, Aladdin). After shaving and disinfection of the operation areas, the skin was cut longitudinally and the periosteum was removed with a blunt instrument to expose the proximal surface of the tibia. Under the irrigation of 0.9% NaCl, the implant hole was drilled with a slow rotation. SLA implants and PT implants were in random order placed into the left and right tibias, respectively. The periosteum and skin were sutured postoperatively. Penicillin (400,000 U/d) was intramuscularly injected into the rats instantly.

After implantation of 10 min or 2 h, a total of 36 rats were sacrificed with a lethal dose of the anesthetic. All implants were screwed out in an anticlockwise direction and gently rinsed with 1 mL PBS 3 times. Then rinsing solution of four implants was collected and centrifuged at 1,000 *g* for 15 min at 4°C. The supernatant was stored at −80°C for further experiments. Implants were fixed with 2.5% glutaraldehyde or 4% PFA for further observation.

### 2.10 SEM of clot network structure

Scanning electron microscopy (SEM, Zeiss Gemini SEM 300, Germany) was used to observe the structure of the blood clot network. Implants retrieved at each time point were fixed in 2.5% glutaraldehyde for 30 min, followed fixed by 2% osmium tetroxide for 20 min, both at room temperature (RT). After dehydration using an ascending series of ethanol from 30% to 100% and critical point drying, the surface of samples was gold-palladium sputter-coated and then observed by SEM. Experiments were performed three times and five images per sample were used for observation.

### 2.11 Immunofluorescence staining of deposited blood components

LCSM was performed to visualize the deposited blood components. After fixation with 4% PFA, a rinse with PBS, permeabilization with 0.5% Triton X-100 for 5 min at RT. SLA and PT implants were blocked in bovine serum albumin (BSA) for 15 min, and then separately incubated with anti-human fibrin antibody (GeneTex, GTX19079, United States) for fibrin, monoclonal anti-integrin α2β-CD41 (Santa, sc-365938, United States) for platelets, monoclonal antiCD62p-P-selectin (Santa, sc-8419, United States) for activated platelets and monoclonal antiCD14 (Santa, sc-515785, United States) for monocytes/macrophages at 4°C overnight. Alexa Fluor 488-conjugated goat anti-mouse secondary antibodies (Abcam, ab150113, Britain) were used for the detection. The F-actin and nuclei were labeled with rhodamine-phalloidin and DAPI. Stained and mounted titanium implants were imaged and analyzed with an LCSM. A confocal z-stack was used to quantify the conformation and components of clots.

### 2.12 ELISA of the coagulation cascade, complement system, and platelets activation

The collected supernatant stored at −80°C was subsequently analyzed by using enzyme-linked immunosorbent assay (ELISA) kits Rat thrombin-antithrombin Complexes (TAT, CUSABIO, CSB-E08432r), Complement Fragment 5a (C5a, CUSABIO, CSB-E08513r), soluble P-selectin (P-selectin or CD62p, CUSABIO, CSB-E07339r) to detect TAT complexes, complement factor, and activated platelets, respectively. ELISAs were performed according to the manufacturer’s protocols.

### 2.13 Specimen harvest for RNA-sequencing

Tibia implantation model was established in C57BL/6 male mice aged 6–8 weeks. The operation was the same as that of SD rats. After 3 days of implantation, tibiae (the proximal metaphysis, cylinder) with implants inside were harvested by cutting off the skin and removing soft tissues. Total RNA was extracted from dissociated cells using a Trizol reagent kit. RNA quality was assessed on an Agilent 2100 Bioanalyzer (Agilent Technologies, United States) and checked using RNase-free agarose gel electrophoresis. After total RNA was extracted, the enriched mRNA was fragmented into short fragments and reverse transcribed into cDNA with random primers. Then the cDNA fragments were purified with a QiaQuick PCR extraction kit (Qiagen, Netherlands), end-repaired, poly(A) added, and ligated to Illumina sequencing adapters. The ligation products were size selected by agarose gel electrophoresis, PCR amplified and sequenced using Illumina HiSeq2500. Differentially expressed genes (DEGs) RNAs differential expression analysis was performed by DESeq2 (Love et al., 2014) software between two different groups. The genes with the parameter of false discovery rate (FDR) below 0.05 and absolute fold change ≥ 2 were considered DEGs.

### 2.14 Statistical analysis

Quantified data were presented as mean ± standard deviation (SD) of at least three independent experiments. All data were analyzed by GraphPad Prism 9 (GraphPad Software Inc., United States), and the statistical significance between different groups was determined by two-way ANOVA. Asterisks denoted statistical significance as follows: *p < 0.05, **p < 0.01, ***p < 0.001, ****p < 0.0001.

## 3 Results

### 3.1 Surface characterization

**FIGURE 1:**
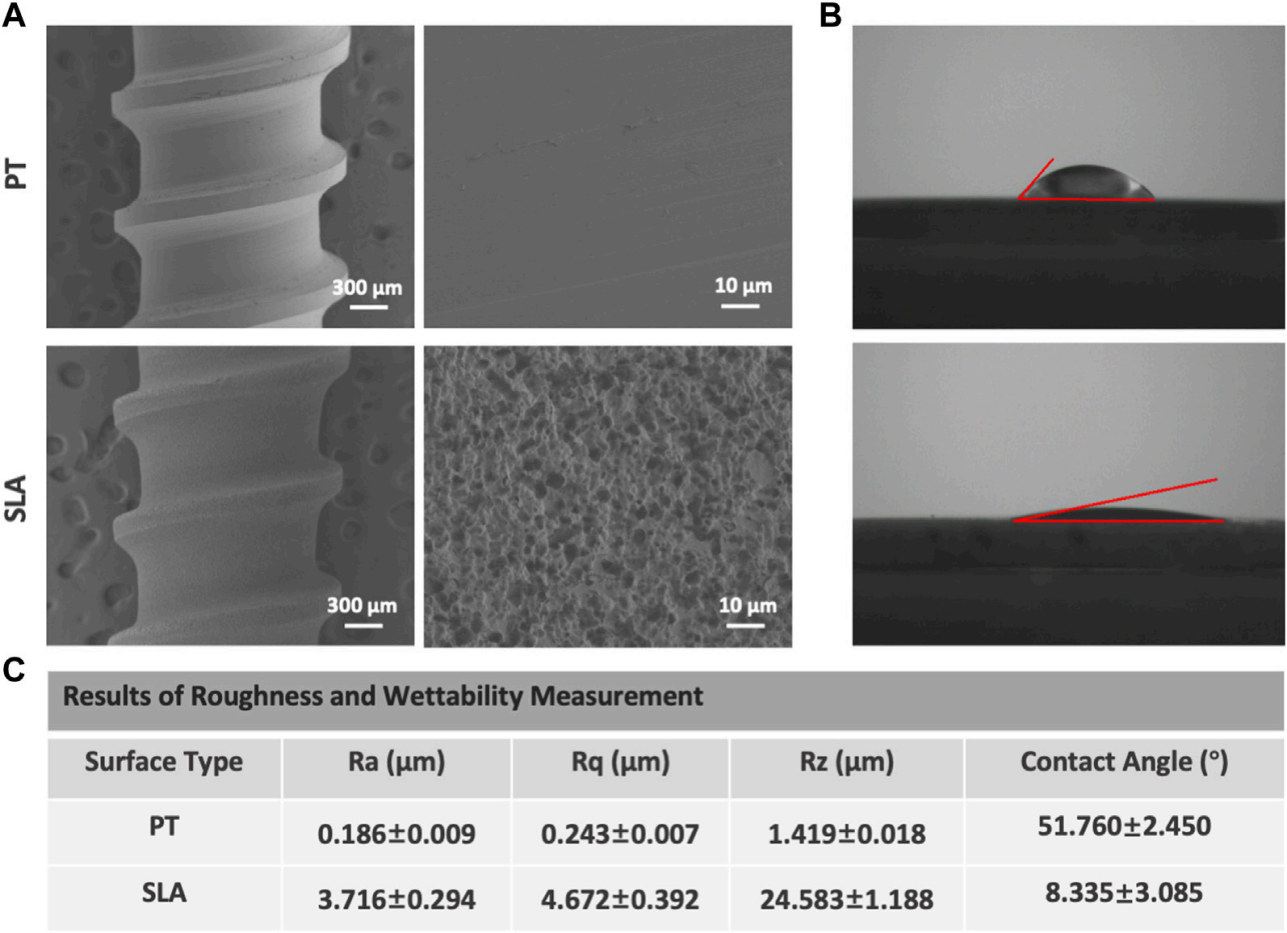
Representative SEM and water CA photographs of PT and SLA surfaces. **(A)** Surface topography was observed by SEM under different magnifications of PT and SLA titanium surface. **(B)** The hydrophilicity of PT and SLA surfaces was assessed by the measure of water contact angle. **(C)** The analysis for the characteristic parameter of surface roughness and hydrophily. Each experiment was repeated three times.

Scanning electron micrographs of titanium surfaces are shown in Figure 1A. The PT surface did not show any microtextures. After sandblasting and acid etching, the micro rough surface is characterized by numerous heterogeneous ridges and concavities with equally distributed honeycomb-like texture with 1–2 micron scale pits. At higher magnification, the disorganized micropore arranged 5–15 μm large holes outside and 100–200 nm small holes inside. Further to assess the hydrophobicity, we measured contact angles to explore the adsorption capacity of titanium surfaces to blood components. The water contact angles were 49.31° for PT surface and 11.42° for SLA surface in static condition displayed in Figure 1B The mean surface roughness (Ra) was 0.186 and 3.716 μm for the titanium surfaces of the PT and SLA groups, respectively (Figure 1C). It was indicated that SLA implants have a rougher surface with micro-size particles and higher hydrophilicity than PT implants.

### 3.2 Cell adhesion and proliferation on pre-incubated surfaces

**FIGURE 2:**
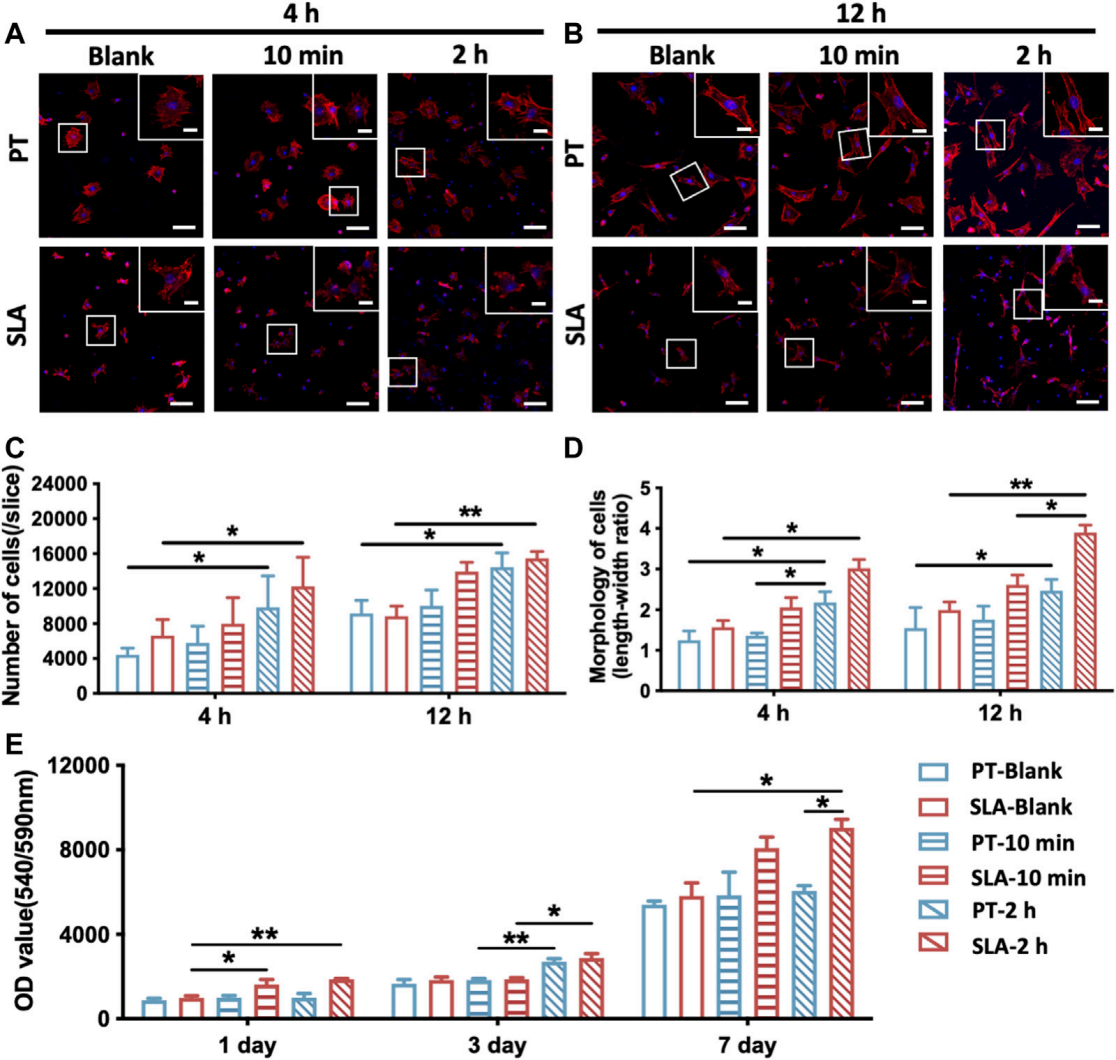
Adhesion and proliferation of BMSCs on PT and SLA surfaces. **(A,B)** LCSM images of BMSCs stained with F-actin (red) and nuclei (blue), cultured after 4 and 24 h on PT and SLA surfaces with or without blood incubation (Scale bars: 200 μm; Inserts: 25 μm). **(C)** Semi-quantitative analysis of the number of adhered cells on surfaces after 4 and 12 h culture. Data represent means ± S.D. **(D)** Quantitative analysis of the ratio of the longest and shortest diameters of the adherent cells on surfaces after 4 and 12 h culture. **(E)** AlamarBlue assay to assess the proliferation of BMSCs grown on different surfaces with or without blood incubation. *p < 0.05, **p < 0.01, ****p < 0.0001.

*In vitro*, the initial cell adhesion and morphology of cells on PT and SLA surfaces with or without blood incubation were observed by staining the nuclei and F-actin 4 or 12 h after cell culture. As shown in Figures 2A, B, the number of adherent cells increased over blood-incubated time on both titanium surfaces, especially in 2 h-pre-incubated surfaces (Figure 2C). BMSCs cultured on the PT and SLA surfaces showed no significant difference in the number of the initially attached cells but showed a difference in morphology. There are a large number of slender BMSCs on the surface of SLA. In contrast, BMSCs adhered to the PT surfaces were rounder with shorter parapodia. After blood incubation, the morphology of cells was significantly extended compared with that of the un-incubated group, especially after 2 h pre-incubation (Figure 2D). This phenomenon indicates that the BMSCs adhered to the SLA surfaces are more active and have the potential for further differentiation.

Besides, cell proliferation on the pre-incubated surfaces significantly increased after being cultured for 3 or 7 days (Figure 2E). The data showed that the proliferation rate of BMSCs on the pre-incubated SLA was significantly higher than that on the control surfaces, especially after 2 h pre-incubation. These results revealed that SLA surfaces with clot networks and blood components might conduce to BMSCs proliferation.

### 3.3 Effects of titanium surface with clot networks and blood-derived conditioned medium on cell migration and recruitment

**FIGURE 3:**
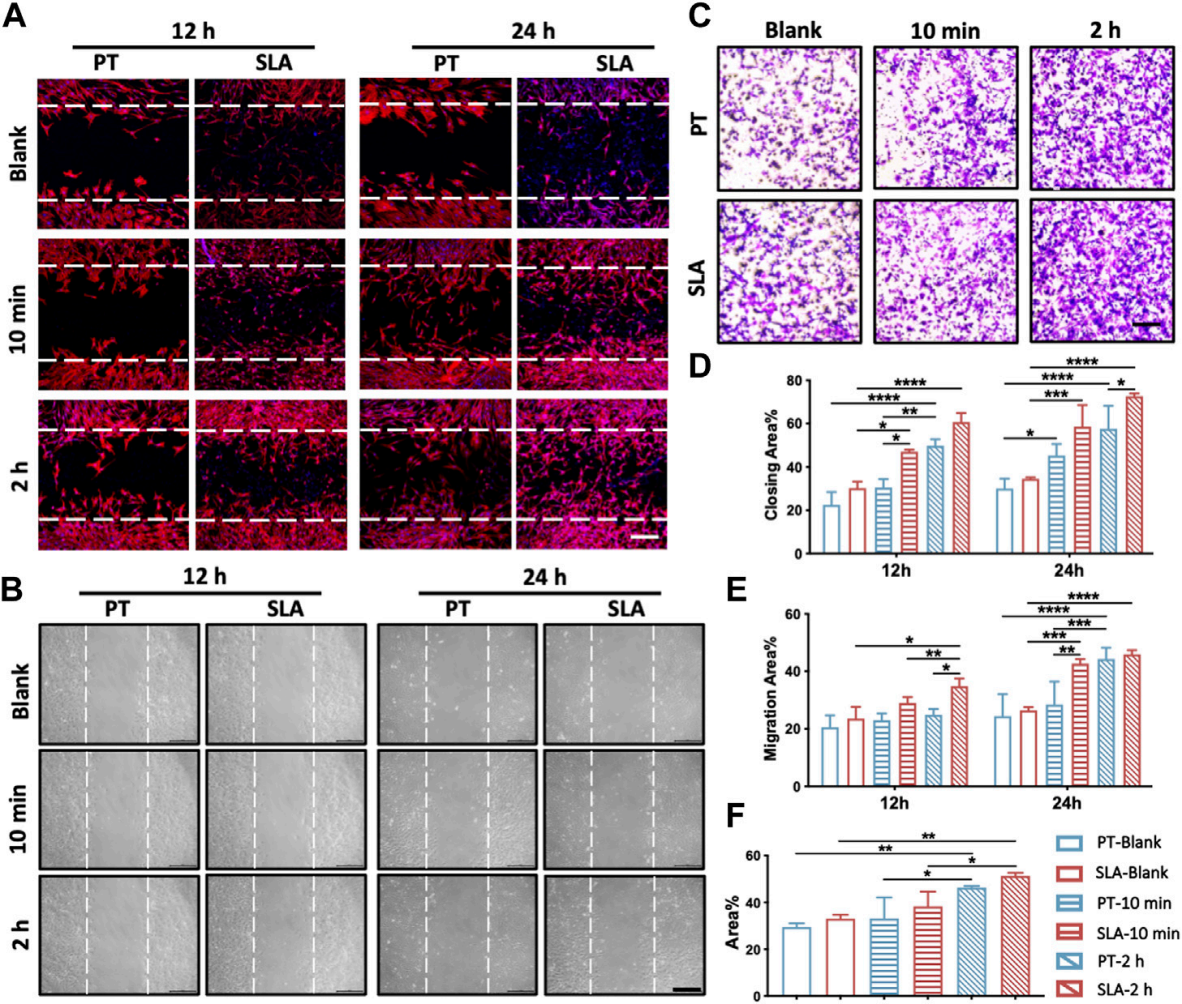
Migration and recruitment of BMSCs on various surfaces. **(A)** Representative images of the scratch wound assay. BMSCs were cultured on the control, 10 min- or 2 h-pre-incubated titanium surfaces in normal media. Scale bars: 250 μm. **(B)** Representative images of the wound healing assay with conditioned media. BMSCs were treated with the extract of control, 10 min- or 2 h-pre-incubated surfaces, respectively. Scale bars: 200 μm. **(C)** Representative images of the transwell assay with conditioned media. Scale bars: 200 μm. **(D)** Quantitative comparison of migratory cells among different groups. **(E)** The proportion of cell migration into the wound under conditioned media was quantified as the level of wound healing. **(F)** Quantitative comparison of recruiting cells with conditioned media among different groups. These experiments were repeated three times with similar results. *p < 0.05, **p < 0.01, ***p < 0.001, ****p < 0.0001.

To further determine whether various titanium surfaces with whole blood incubation could affect cell migration and recruitment, the scratch wound-healing assay and the transwell assay were performed (Figure 3). The migratory capacity of BMSCs was gradually enhanced after being cultured on pre-incubated titanium surfaces via the scratch wound-healing assay. After 24 h, the wound closure rate of BMSCs seeded on with 2 h-pre-incubated SLA surface was 72.3% ± 2.3%, which led to an approximately 0.83-fold increment than PT counterparts (39.6% ± 4.2%, Figures 3A, D). To further exclude the influence of surface topography, the conditioned medium derived from the pre-incubated surfaces was used to culture BMSCs seeded on culture plates in the wound healing assay. No statistically significant difference in the migration area proportion was observed between the 10 min-pre-incubated and the control group at 12 h. Nevertheless, the cell mobility displayed an increased tendency at 24 h when treated with the conditioned medium from the pre-incubated SLA surfaces in contrast to the PT counterparts (Figures 3B, E).

Transwell assay was conducted to further verify the effect of the paracrine effect of blood components on the recruitment of BMSCs. As shown in Figures 3C, F, the conditioned medium from 2 h-pre-incubated SLA and PT surfaces respectively led to 0.55-flod and 0.57-fold increment than control group in cell mobility. Consistent with the scratch wound experiments, the proportion of recruited BMSCs rose sharply with extended blood-incubation time, from 33.08% to 51.33% in the SLA group.

The above results supported that different titanium with fresh clots could facilitate the mobility of BMSCs. Moreover, blood components release factors that stimulate cell migration and enhance the recruitment of BMSCs, which might contribute to wound healing.

### 3.4 The osteogenic differentiation of BMSCs cultured on the pre-incubated surfaces

**FIGURE 4:**
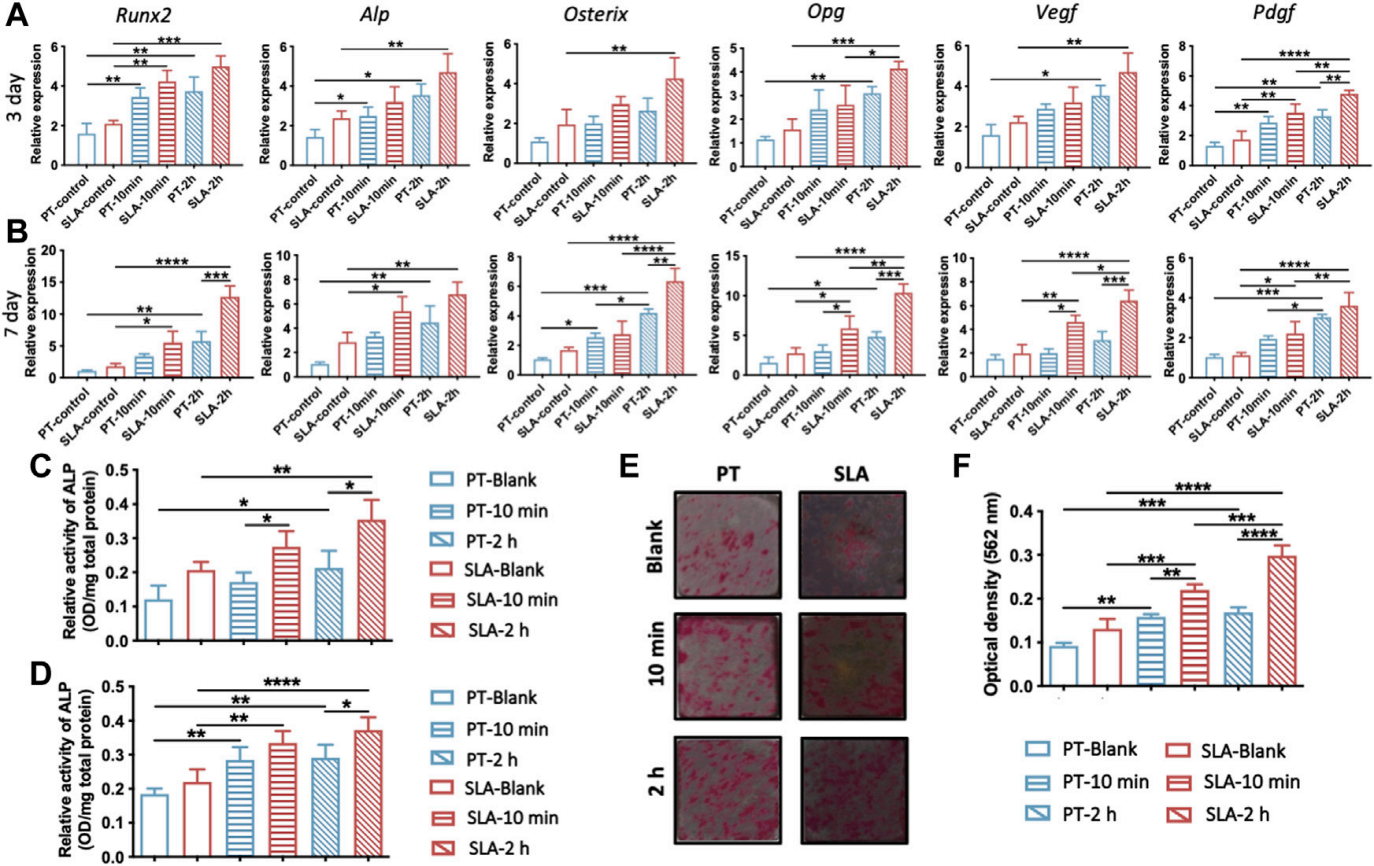
Osteogenic and angiogenic differentiation of BMSCs on various titanium surfaces. **(A,B)** Expression levels of osteogenic genes (ALP/Runx2/Osterix/Opg) and angiogenic genes (Vegf/Pdgf) in BMSCs cultured on titanium surfaces with or without blood incubation were evaluated on day 3 **(A)** and day 7 **(B)**. **(C,D)** Quantitative analysis of ALP activity of BMSCs after 4 days **(C)** and 7 days **(D)** culture, respectively. **(E)** Representative photos of Alizarin Red S staining of calcium nodules on PT and SLA surfaces on the 21st days. **(F)** Quantitative analysis of calcium nodules by cetylpyridinium chloride solution. Error bars represent the SD of three independent experiments. *: *p* < 0.05, **: *p* < 0.01, ***p < 0.001, ***p < 0.0001.

To verify the effect of blood clots on the osteogenic differentiation of BMSCs, the expression level of osteogenesis-related and angiogenesis-related genes were evaluated by RT-qPCR after 3 or 7 days of culture (Figures 4A, B). The expression of *Osterix* and *Opg* in the SLA group was higher than that in the PT group without blood incubation on the seventh day. On the pre-incubated surfaces, the transcriptional levels of *Runx2*, *Alp*, *Osterix*, and *Opg* showed an upward trend and were highest on the 2 h-pre-incubated SLA surface. A marked elevation of genes expression in cells cultured on pre-incubated disks than those of un-incubated surfaces. Especially on day 7, the mRNA levels of the *Vegf* and *Pdgf* on 10 min- and 2 h-pre-incubated SLA surface were significantly increased compared with the ctrl group.

Given that osteogenic differentiation in gene level was upregulated by blood clots, we further evaluated the ALP activity and bone calcium nodule formation of BMSCs. BMSCs cultures on the pre-incubated surfaces displayed higher ALP activity than the blank group, especially on 2 h-pre-incubated surfaces (Figures 4C, D). In addition, the ALP activity of BMSCs cultured on the pre-incubated SLA surfaces was significantly higher than that on the PT counterparts at both time points. The calcium nodule formation on titanium surfaces was stained and quantified by cetylpyridinium chloride solution (Figures 4E, F). 10 min- and 2 h-pre-incubated SLA surfaces showed 1.67 times and 2.27 times alizarin red than the control group, respectively. Meanwhile, the pre-incubated PT surface showed the same trend. It hinted at the promotive role of blood clots on the osteogenic differentiation of BMSCs.

### 3.5 Characterization of blood clot features on titanium surfaces

**FIGURE 5:**
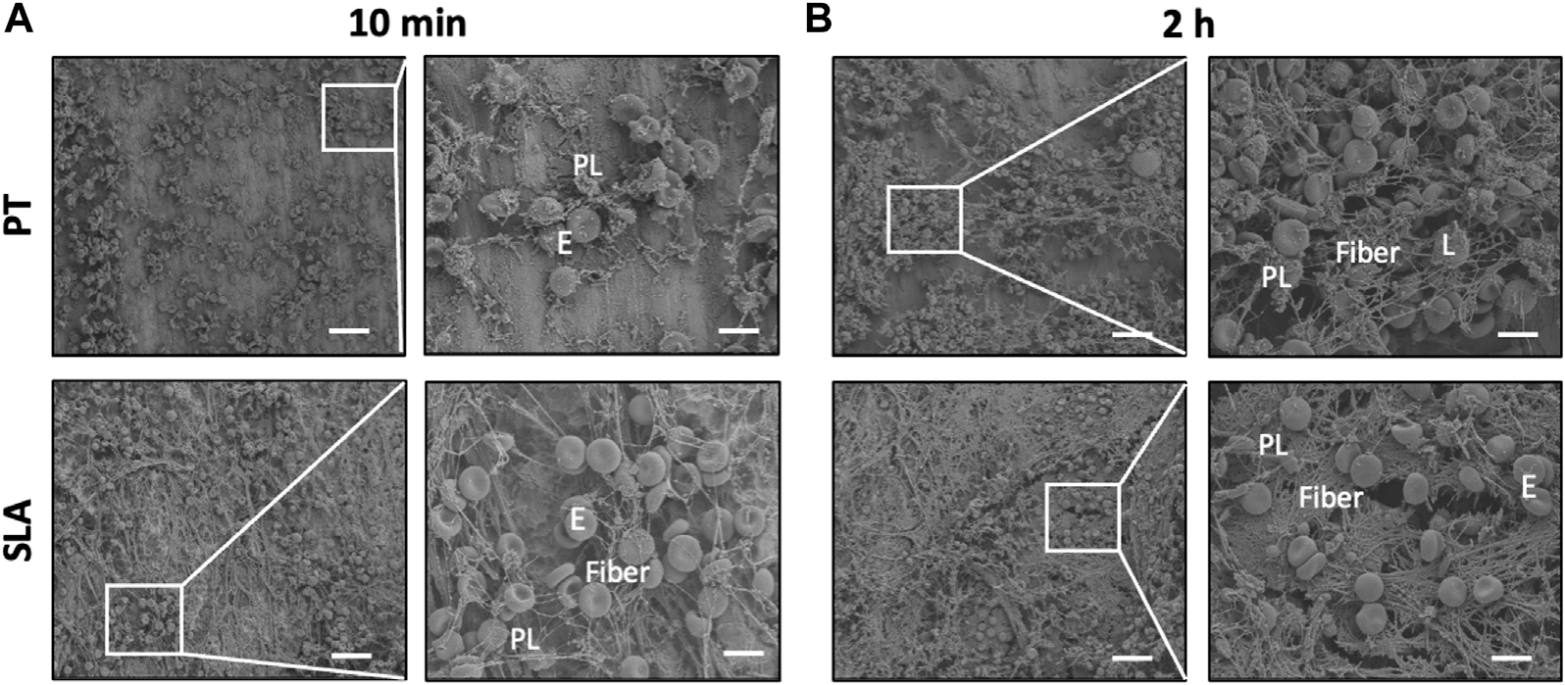
Characterization of blood clot features on titanium surfaces. **(A,B)** Scanning electron micrographs of adhering blood clots and entrapped blood components. Representative SEM images showed the adhering blood clots with entrapped platelets and cells on the surface of PT and SLA implants after 10 min **(A)** and 2 h **(B)** implantation. Scale bars: 80 μm; Inserts: 20 μm. E, erythrocytes, PL, platelets, and L, leukocytes.

Representative SEM images of blood clot characteristics on different implant surfaces *in vivo* were presented in Figure 5. With the prolongation of time after implantation into tibia, a stronger blood coagulation response was observed, showing a tightly-packed and hierarchical structure with filamentous fibers, more entrapped platelets, and blood cells. Moreover, distinct morphological differences between the clots formed on the different titanium surfaces. The scattered blood components stick to the PT surface, with only a cluster of platelets and a few fibers loosely interwoven. While on the SLA surface, more platelets and fibers adhered within 10 min. After 2 h, holes and path-like structures within a thick layer of blood clot were formed.

### 3.6 Interaction of blood components with titanium surfaces after implantation

**FIGURE 6:**
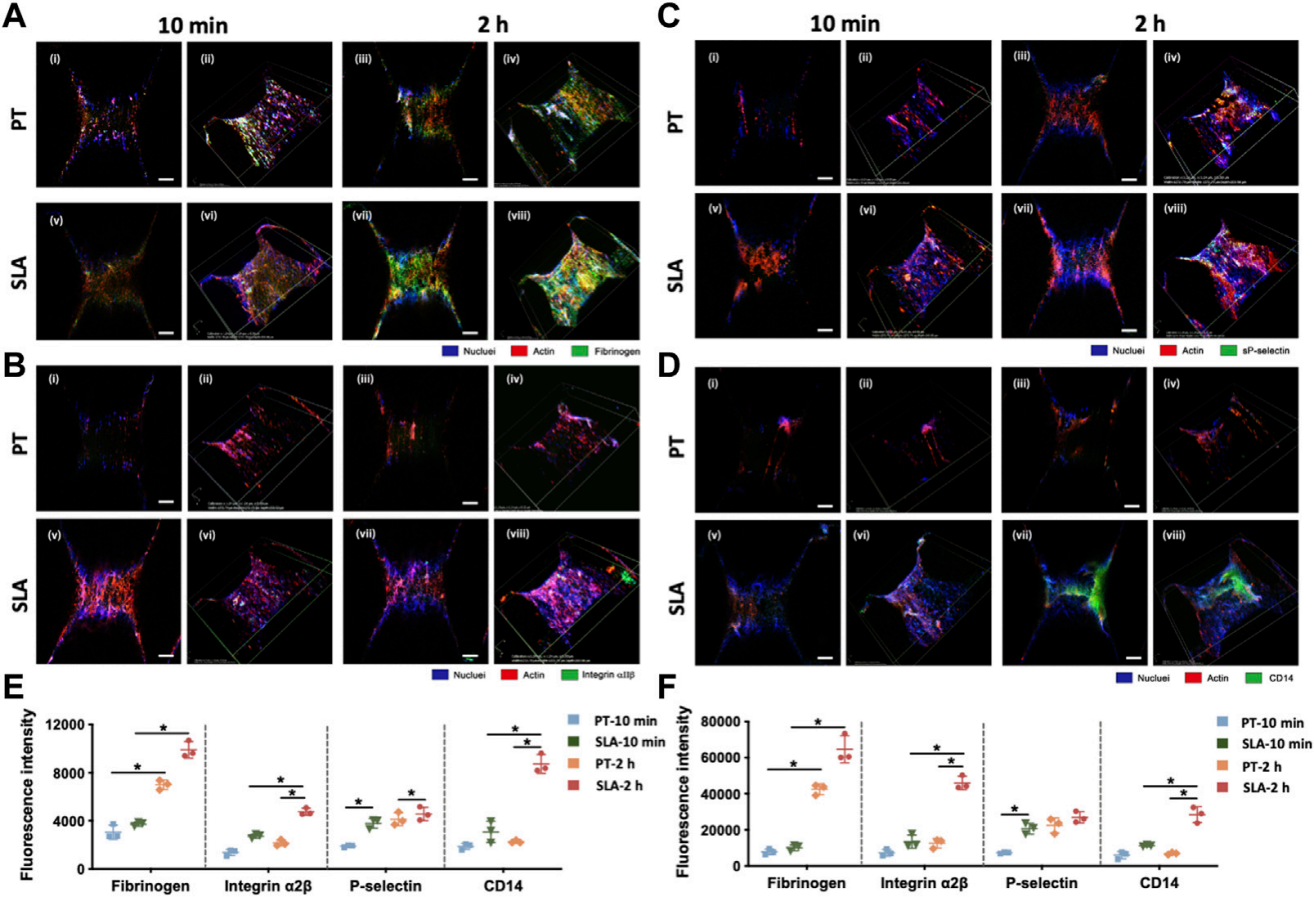
Laser confocal scanning micrographs of blood clot composition and features on PT and SLA implant surfaces. **(A–D)** The two-dimensional (i, ii, v, vi) and z-stack projective (iii, iv, vii, viii) fluorescence images were shown. Surfaces stained with DAPI for nuclei (blue), with rhodamine-phalloidin for F-actin (red) and with **(A)** Anti-fibrinogen for fibrin; **(B)** Integrin α2β for clotting response; **(C)** P-selectin for activated platelets; **(D)** CD14 for monocyte-macrophage cell linage (Scale bars: 200 μm). **(E,F)** The fluorescence intensity analysis for 2D **(E)** and 3D **(F)** z-stack images. Images represent 3 biological replicates. *p < 0.05, **p < 0.01.

To clarify the features of blood clots formed on the titanium implant surfaces *in vivo*, LCSM was used to characterize fibrinogen, activated platelets, and monocytes/macrophages after staining with specific antibodies. Immunofluorescence micrographs and projection of z-stacks of images rebuilt the structure of implants, and semi-quantitatively analyzed (Figure 6). The SLA surfaces induced the formation of thicker blood clots with a higher density of a fibrous structure and trapped blood components.

The conformation of cross-linked fibrinogen was different from the macroscopic images, which was the central building block of the blood clots (Figure 6A). Immunofluorescence density of fibrinogen in confocal micrographs substantially increased after 2 h than 10 min. Furthermore, fibrinogen adsorbed more to the SLA surface than to the PT surface. Macroscopic projection of confocal z-stacks also showed a well-proportioned fibrin network attaching to SLA surfaces while only sparse fibrin fibers formed on PT surfaces.

Platelet was immune-stained with platelet membrane glycoprotein integrin α2β (CD41) and platelet activation-dependent granular membrane protein (P-selectin). Figures 6B, C shows that the mean fluorescence area coverage with CD41-positive and P-selectin-positive platelets was higher on the SLA surfaces than on the PT surfaces, and the intensity value steadily rose over time. In addition, flow cytometry was supplemented to evaluate the activated platelets surrounding the implant surfaces (Supplementary Figure S1A). Results showed that the percentage of activated platelets increased with the extension of implantation time (Supplementary Figure S1B). There was no significant difference between PT surface and SLA surface at 10 min, but SLA surface significantly activated platelets compared with PT surface after 2 h. Overall, current data suggested that the activation degree of platelets had a positive correlation with time and was influenced by biomaterial properties.

Due to the abundance of monocytes in the whole blood and the pivotal role of the monocyte-derived macrophages in inflammation and osteointegration, CD14 was used as a marker for monocytes and macrophages to count the cell number (Figure 6D). Little monocytes were adsorbed on the implant surface within 10 min after implantation. After 2 h implantation, numerous CD14-positive cells within blood clots attached to the SLA surface, in contrast to a dramatically lower fluorescence intensity on the PT surfaces.

### 3.7 Activation of the blood coagulation cascade, the complement system, platelets, and cellular responses *in vivo*

**FIGURE 7:**
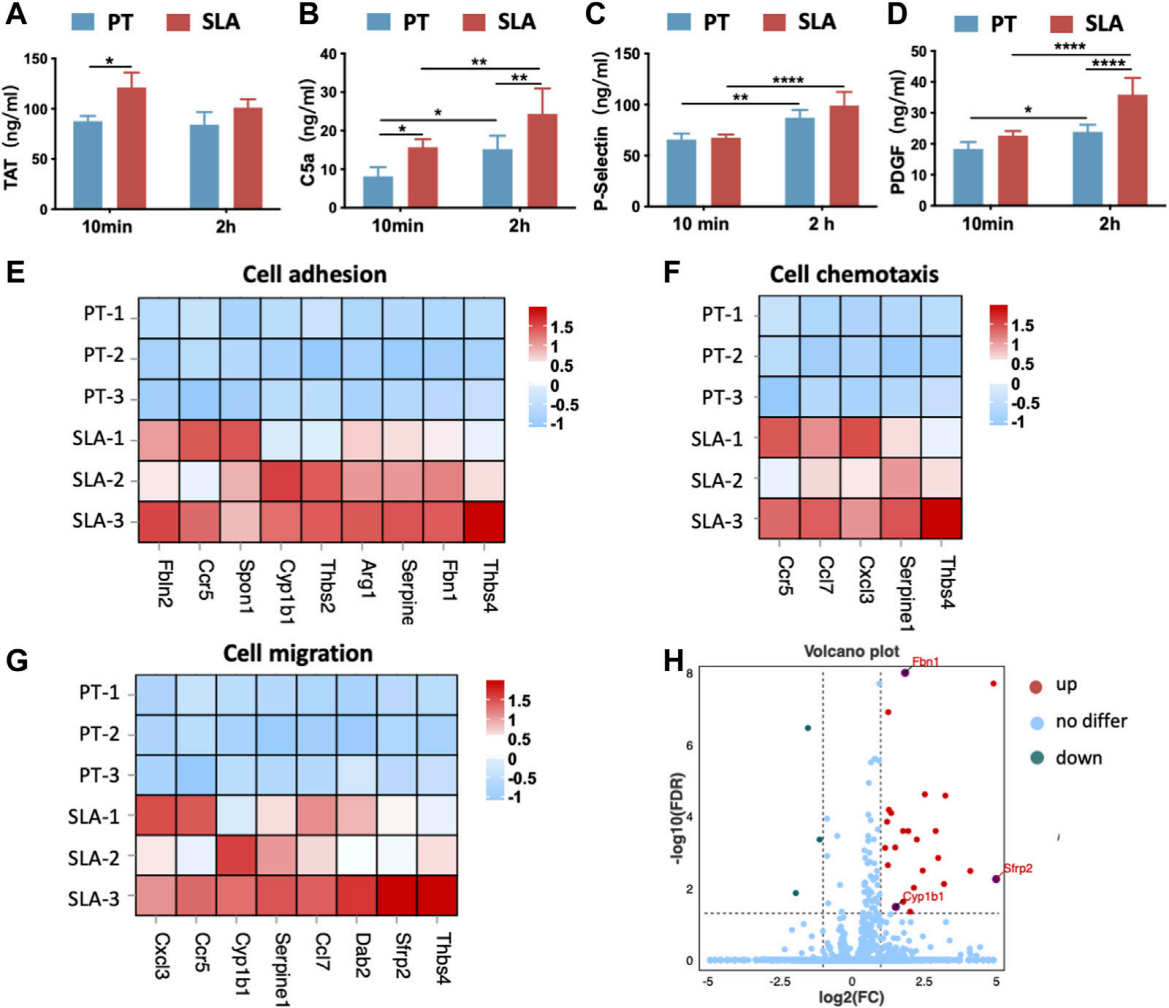
The activation of cellular response after implantation *in vivo.* **(A–D)** The concentration of TAT complexes **(A)**, C5a **(B)**, P-selectin **(C)**, and PDGF **(D)** was detected by ELISA. Error bars represent the SD of three independent experiments. *: *p* < 0.05, **: *p* < 0.01, ****: *p* < 0.0001. **(E–G)** Heatmap of genes expression in cell adhesion (GO:0007155), cell chemotaxis (GO:0060326), and cell migration (GO: 0016477). **(H)** Volcano plot showing the DEGs between two implant materials. SLA implants upregulated the expression of extracellular matrix organization-related genes (*Sfrp2*, *Cyp1ba*, *Fbn1*) when compared with the PT implants.

The activation of titanium surfaces on biological processes *in vivo* including blood coagulation, complement system, and activated platelets was monitored by ELISA against TAT complexes, the anaphylatoxin C5a, the P-selectin, and PDGF (Figures 7A–D). Compared with the PT surface, the SLA surfaces induced a significant increase in TAT and C5a concentrations at both time points. The concentration of TAT slightly decreased over time throughout the implantation period. It was indicated that both types of surfaces increased C5a concentration over time. Besides, an increasing trend was observed in P-selectin and PDGF concentrations after 2 h in contrast to the 10 min group, but the difference in P-selectin between the two surfaces was not statistically significant. After 2 h, the concentration of PDGF on the SLA surface was significantly higher than that on the PT surface. Statistically, P-selectin concentration showed surface properties with little influence, but implantation time showed a significant effect. However, PDGF was affected by both time and surface properties.

Further, bone tissue RNA-sequencing (RNA-seq) analysis for samples harvested 3 days (*n* = 3) after implantation. DEGs analysis showed that the implantation of SLA implants induced a higher expression level of related genes in cell adhesion, cell chemotaxis, and cell migration when compared with PT implants (Figures 7E–G). This trend was consistent with *in vitro* results. In addition, SLA implant upregulated the differentially expressed genes associated with extracellular structure organization including *Sfrp2*, *Cyp1ba*, *and Fbn1* (Figure 7H). Thus, SLA implants initiated stronger platelet activation and more antithrombin formation, as well as promoting cell adhesion and migration than PT surfaces.

## 4 Discussion

Blood clots that form immediately following bone implantation play a vital role in the subsequent healing process (Wang et al., 2017). The modulation of blood clot structure within the implant surface could lead to the development of multifunctional scaffolds that mimic the natural structure to accelerate bone regeneration (Liang et al., 2021). How blood clots as mediators between the biomaterial surface and the osteogenic differentiation of BMSCs are worth further exploring. The current study demonstrated that titanium surface characteristics directly affect platelet activation and clot formation immediately after implantation, and clot features steer the recruitment and osteogenic potential of BMSCs.

The blood components and adsorption proteins act as bridging scaffolds between the implant surfaces and cells. Plasma-derived proteins, platelet-rich fibrin, or blood-prefabricated materials were used as an effective strategy to enhance bone repair and regeneration (Kopf et al., 2015b; Liang et al., 2021; Ren et al., 2022; Wu et al., 2022). The number of cells adhering and recruitment increased on the blood-pretreated surfaces (Yang et al., 2016). Interestingly, compared with the control group, BMSCs tended to extend branched projections from the cell body while showing a spindle-shaped spread after blood incubation. Ordinarily, osteogenic cells protrude more filopodia and possess stronger migratory and osteogenic potential (Kopf et al., 2015a; Schar et al., 2015). This finding supports that the surface has a blood clot and interacts with blood components are crucial in tailoring the cellular response.

The surface with clot networks is crucial for withstanding detachment forces, thus providing a migration pathway for the osteogenic cells to reach the implant surface (Arvidsson et al., 2007). The blood-prefabricated implant materials exhibit superior performance in early cell recruitment via releasing potent migration factors such as CINC-2a, IL-2, L-selectin, MCP-1, PDGF-AA, and VEGF (Lam et al., 2015; Bruekers et al., 2016; Eriksson et al., 2019). Consistent with previous studies, current results showed that migration and recruitment are improved not only on pre-incubated surfaces but also in the use of conditioned mediums. The clot networks can release cytokines and chemokines to modulate the interaction of BMSCs with the titanium surfaces and the cytokines released by the clots on the SLA surface showed stronger chemotaxis at two-time points *in vitro*. The osteogenic differentiation potential of BMSCs is enhanced after cultured on the pre-incubated surfaces over time. Thus, after interacting with blood clots on the surface, BMSCs exerted better recruitment, mobility, and osteogenic differentiation at the early stage of the bone healing process.

The blood clot acts as a “natural scaffold” that forms when the extrinsic coagulation pathway is activated. This pathway relies on a complex interaction between three major components, coagulation factors, activated platelets, and blood cells. The composition and structure of clots such as fibers thickness, porosity, number of branching points, and the strength of the adhesion of the fibrin clots depend on the surface properties (Chen et al., 2014; Chen et al., 2015). SLA surfaces due to the more intense contact activation and thrombotic characteristics, resulting in clots with thicker fibrin fibers, more platelets, and a stable porous fibrin scaffold. The initial adhesion of platelets binds to implant-surface-adsorbed fibrinogen by integrin α2β (Broberg et al., 2002). Rough surface microtopography would exhibit an increased surface area and a resultant augment in fibrinogen absorption, which explains the increase in platelet adhesion, activation, and aggregation (Collignon et al., 2017). Evidence showed that fibrinogen provided a temporary microenvironment conductive to regeneration, which led to bone repair after 8 weeks of implantation (Vasconcelos et al., 2016). Fibrinogen also modulates the activity of monocytes and macrophages through knock-on stimulatory events (Zuchtriegel et al., 2016; Kaur et al., 2017). Besides, the platelet-directed guidance of leukocyte’s extravasation to implant surfaces is an essential step in the recruitment process of immune cells (Lam et al., 2015). Platelet exposure to P-selectin leads to ERK 1/2 MAPK-dependent conformational change in leukocyte integrins and promotes the continuing extravasation of neutrophils and monocytes to the implant site (Zuchtriegel et al., 2016). Afterward, the increasing number of monocytes/macrophages (CD14 positive cells) entrapped on the SLA surface shows beneficial phagocytic activity and bioactive molecules release, which are necessary for cleaning a wound site (Holmes et al., 2018). The SLA surface enhances the adhesion thickness of fibrin clots and the complexity of the three-dimensional network structure. Successful tissue healing has been shown to depend on the initial fibrin-clot formation within the defect site. Evidence suggests that the removal of a clot causes delayed fracture healing and subcutaneous implantation of a hematoma results in ectopic bone formation (Kolar et al., 2011; Wei et al., 2018).

Our study further revealed that roughness and hydrophilicity could effectively affect the structure of blood clots and the adsorption of proteins *in vivo* which is in line with the other *in vitro* studies (Fan et al., 2017; Sangkert et al., 2022). Park and colleagues demonstrated that the complexity of the surface significantly increased platelet attachment. The hydrophilic surface shows a higher platelet binding and more intense contact coagulation cascade. Rougher and hydrophilic surfaces induce greater and earlier platelet release in subsequent macrophage inflammatory cytokine responses, thereby immunomodulating the ability to bone regeneration (Zivkovic et al., 2021).

Native titanium stimulates the generation of thrombin in the blood due to contact activation *in vivo* (Zabczyk et al., 2019). Thrombin cleaves fibrinogen into fibrin monomers, which then polymerize into the initial fibrin clot, which occurs in response to the dynamic changes in thrombin concentration (Hulshof et al., 2021). Based on TAT concentration implied that the SLA surface had strong thrombosis characteristics. Thrombin inhibitor participates in BMSCs behaviors and promotes osteogenesis via canonical Wnt signaling pathway (Song et al., 2021). Furthermore, an increasing trend indicated that the activation of the complement system and platelets are time-dependent. With incubation time extended from 10 min to 2 h, more blood clots with complement factors and activated platelets on the surfaces. Both complement proteins and platelets communicate with each other and facilitate progression into the inflammatory stage of bone healing (Schraufstatter et al., 2009). The anaphylatoxins C5a present in injured tissues induce an inflammatory response and act as a chemokine for MSCs and regulate their behavior (Holmes et al., 2018).

In this study, an *in vitro* blood incubation model was adopted to simulate the immediate contact of implants *in vivo*. But heparin sodium is not present in the body, which is a limitation of the experimental design. The experimental results excluding the effect of heparin sodium at the same concentration on BMSCs are shown in Supplementary Figure S2. In addition, *in vivo* studies lack direct observation of BMSCs, which is the direction of our further research. A lot of work is still worth studying in the future.

In summary, this study reveals the interactions between clot features and the behaviors of BMSCs on titanium surfaces, providing a novel research idea for biomaterial design. SLA surfaces induced stronger platelet activation and more antithrombin formation, as well as more complex stereoscopic clot features than PT surfaces. In turn, the surface with blood clots promotes the recruitment and osteogenic differentiation of BMSCs. Given the physiological role and tailoring potential of the blood clots, it is crucial to understand the properties of blood clots formed on the implant surface in order to develop improved biomaterials for better osteogenesis.

## Data Availability

The original contributions presented in the study are publicly available. This data can be found here: https://www.ncbi.nlm.nih.gov/bioproject/PRJNA924055/.
