# Supplementary material for: Titanium surface interacting with blood clot enhanced migration and osteogenic differentiation of bone marrow mesenchymal stem cells
Source: Front Bioeng Biotechnol. 2023 May 16;11:1136406. doi: 10.3389/fbioe.2023.1136406 (PMC10227579; doi:10.3389/fbioe.2023.1136406)
Supplement: Supplementary file 1 [file Table1.DOCX]

**Supplementary information:**

**Table 1. Primer sequences used in real-time qPCR**

| Gene | Forward primer sequence (5’-3’) | | Reserve primer sequence (3’-5’) |
| --- | --- | --- | --- |
| Gapdh | GGCACAGTCAAGGCTGAGAATG | ATGGTGGTGAAGACGCCAGTA | |
| Alp | CATCGCCTATCAGCTAATGCACA | ATGAGGTCCAGGCCATCCAG | |
| Runx2 | CCATAACGGTCTTCACAAATCCT | TCTGTCTGTGCCTTCTTGGTTC | |
| Opg | ATCCCAAGGGGTTTCAGATTGG | CCGCGCCTCCGCACCCTGTTC | |
| Osterix | CACCCATTGCCAGTAATCTTCGT | GGACTGGAGCCATAGTGAGCTTCT | |
| Vegf | TGGTCTACCGTCCGGGAATC | GAGTCTTGGGACGCATGGTG | |
| Pdgf | GTGCCGCTGAGTTCGTCCTTC | GCTGAGGCGTTGACCACTTCC | |
