# Supplementary material for: Titanium surface interacting with blood clot enhanced migration and osteogenic differentiation of bone marrow mesenchymal stem cells
Source: Front Bioeng Biotechnol. 2023 May 16;11:1136406. doi: 10.3389/fbioe.2023.1136406 (PMC10227579; doi:10.3389/fbioe.2023.1136406)
Supplement: Supplementary file 2 [file Table2.DOCX]

**Supplementary information:**

**Supplementary figure 1:** **Activated platelets were detected by flow cytometry.**

(A) Flow cytometry analysis of P-selectin-positive platelets at 10 min and 2 h after implantation of PT implants and SLA implants. (B) Flow cytometry analysis of the percentage of activated platelets at 10 min and 2 h.

**Supplementary figure 2. The effect of heparin sodium on the proliferation and osteogenic differentiation of BMSCs.**

(A) AlamarBlue assay to assess the proliferation of BMSCs grown on different titanium surfaces with or without heparin (10 IU) incubation. (B) Expression levels of osteogenic (*Alp*, *Runx2*, Sp7) genes in BMSCs cultured on titanium surfaces with or without heparin incubation were evaluated by RT-qPCR after 3 days and 7 days. **p* < 0.05.
